# Definition and quality requirements for stereotactic radiotherapy: consensus statement from the DEGRO/DGMP Working Group Stereotactic Radiotherapy and Radiosurgery

**DOI:** 10.1007/s00066-020-01603-1

**Published:** 2020-03-24

**Authors:** Matthias Guckenberger, Wolfgang W. Baus, Oliver Blanck, Stephanie E. Combs, Jürgen Debus, Rita Engenhart-Cabillic, Tobias Gauer, Anca L. Grosu, Daniela Schmitt, Stephanie Tanadini-Lang, Christos Moustakis

**Affiliations:** 1 Universität Zürich, Klinik für Radio-Onkologie, Universitätsspital Zürich, Zurich, Switzerland; 2 Klinik für Radioonkologie und Strahlentherapie, Universitätsklinikum Köln, Cologne, Germany; 3 Klinik für Strahlentherapie, Universitätsklinikum Schleswig-Holstein, Kiel, Germany; 4 Klinikum rechts der Isar, Klinik für RadioOnkologie und Strahlentherapie, Technische Universität München, Munich, Germany; 5 Klinik für Radioonkologie und Strahlentherapie, Universitätsklinikum Heidelberg, Heidelberg, Germany; 6 Klinik für Strahlentherapie und Radioonkologie, Universitätsklinikum Gießen Marburg, Marburg, Germany; 7 Klinik für Strahlentherapie und Radioonkologie, Universitätsklinikum Hamburg-Eppendorf, Hamburg, Germany; 8 Klinik für Strahlenheilkunde, Universitätsklinikum Freiburg, Robert-Koch-Str. 3, 79106 Freiburg, Germany; 9 Klinik für Strahlentherapie, Universitätsklinikum Münster, Munster, Germany

**Keywords:** Radiosurgery, Stereotactic Body Radiotherapy, SRS, SBRT, Definition

## Abstract

Stereotactic radiotherapy with its forms of intracranial stereotactic radiosurgery (SRS), intracranial fractionated stereotactic radiotherapy (FSRT) and stereotactic body radiotherapy (SBRT) is today a guideline-recommended treatment for malignant or benign tumors as well as neurological or vascular functional disorders. The working groups for radiosurgery and stereotactic radiotherapy of the German Society for Radiation Oncology (DEGRO) and for physics and technology in stereotactic radiotherapy of the German Society for Medical Physics (DGMP) have established a consensus statement about the definition and minimal quality requirements for stereotactic radiotherapy to achieve best clinical outcome and treatment quality in the implementation into routine clinical practice.

## Introduction

Stereotactic radiotherapy today is a guideline-recommended treatment for malignant or benign tumors as well as neurological functional or vascular disorders. Stereotactic radiotherapy has therefore become a standard procedure in the radiation oncology community, which is also used outside of clinical trials and outside of specialized university centers. Despite the highest level of evidence in randomized controlled trials and despite the existence of national and international practice recommendations, there is no generally accepted definition of stereotactic radiotherapy, especially since accurate image guidance has largely replaced stereotactic frames. Similarly, there are no generally accepted and comprehensive quality requirements for stereotactic radiotherapy.

This lack of consensus was identified by the German Society for Radiation Oncology (DEGRO) and therefore the DEGRO board mandated the DEGRO working group for radiosurgery and stereotactic radiotherapy to establish a statement about definition and quality requirements of stereotactic radiotherapy. Previous published guidelines of the DEGRO working group for radiosurgery and stereotactic radiotherapy [1–4], the European Society of Radiation Oncology (ESTRO) [5], the American College of Radiology (ACR), the American Society for Radiation Oncology (ASTRO) [6, 7] and the United Kingdom Consortium for Stereotactic Radiotherapy [8], as well as international literature and national and international recommendations formed the basis. In 2018, a statement about the definition and general quality requirements for stereotactic radiotherapy was established and published by the DEGRO society. This consensus statement was amended to the current version in collaboration with the German Society for Medical Physics (DGMP) working group for physics and technology in stereotactic radiotherapy, taking into consideration the results of an expert review on technological quality requirements for stereotactic radiotherapy [9].

## Definition for stereotactic radiotherapy

There are three types of stereotactic radiotherapy, which differ with regard to the spectrum of indication, fractionation and quality requirements: (1) stereotactic radiosurgery (SRS) as a treatment of intracranial malignant or benign tumors and functional or vascular disorders with one single irradiation fraction, (2) fractionated stereotactic radiotherapy (FSRT) of intracranial malignant or benign tumors and functional or vascular disorders as well as (3) stereotactic body radiotherapy (SBRT) of extracranial malignant or benign tumors and functional or vascular disorders.

Generally, stereotactic radiotherapy is defined as

1. a method of percutaneous external beam radiotherapy, in which
2. a clearly defined target volume
3. is treated with high precision and accuracy
4. with a biologically high radiation dose
5. in one single or a few fractions
6. with locally curative intent.

Details to (1): Stereotactic radiotherapy can be performed using either linear accelerators or dedicated stereotactic radiotherapy devices (e.g., Gamma Knife [Elekta AB, Stockholm, Sweden], CyberKnife [Accuray Inc., Sunnyvale, CA, USA], Edge [Varian Inc., Palo Alto, CA, USA] and Versa HD [Elekta AB]), which fulfill the minimal technological quality requirements as listed below.

Details to (2): The target volume is clearly defined and not characterized by a diffuse infiltration into critical serial organs-at-risk. For tumors, the target volume is confined to the macroscopic tumor and a small surrounding volume of potential microscopic tumor spread.

Details to (3): All substeps of stereotactic radiotherapy are systematically optimized and appropriate quality assurance measures have been implemented to achieve high precision and accuracy of stereotactic radiotherapy. From a clinical perspective, this includes disease staging, the interdisciplinary discussion for indicating stereotactic radiotherapy, the choice of optimal pretreatment imaging with appropriate spatial and temporal resolution for target volumes and organs-at-risk definition and highly conformal radiation treatment planning, frame-based and/or image-guided radiation delivery with active or passive motion management during treatment and close follow-up including dedicated imaging for evaluation of the treatment outcome, preferably by the treating institution. From a medical physics perspective, additional and more sophisticated quality assurance procedures and often tighter acceptance limits are necessary for stereotactic radiotherapy as compared to conventional fractionated radiotherapy.

Details to (4): Due to extreme hypofractionation, radiotherapy doses in stereotactic radiotherapy are biologically at least as high, often higher, as compared to radical radiation therapy doses using conventional fractionation.

Details to (5): Stereotactic radiotherapy doses are delivered in a few (maximum 12) fractions. A risk-adapted adjustment of the fractionation and the total dose based on the volume and location of the target is essential.

Details to (6): The primary goal of stereotactic radiotherapy is long-term local tumor control for cancer therapy or the destruction of cell and/or tissue function for functional or vascular indications with minimal risk of side effects. In most cases, this local tumor control or the destruction of cell and/or tissue function results in a higher-ranking clinical goal, e.g. symptom control or prognosis improvement of the underlying disease.

## Quality requirements for stereotactic radiotherapy

The essential technological and processual quality requirements for stereotactic radiotherapy are summarized in the following. These are to be interpreted as minimal requirements which must be fulfilled to achieve best clinical outcome and treatment quality for stereotactic radiotherapy. For further details on the technological requirements we refer to the expert review of the DGMP working group for physics and technology in stereotactic radiotherapy [9].

### Technological quality requirements

#### (1) Imaging for target volume definition:

The target volume and all organs-at-risk are defined using organ-specific imaging modalities and standardized imaging protocols dedicated for stereotactic radiotherapy procedures. The use of secondary imaging requires accurate registration with the thin-slice planning computed tomography (CT).

#### (2) Patient positioning and target volume localization:

Daily in-room image-guidance and online correction of target position errors using on-board CT, supplementary in-room CT or stereoscopic X‑ray is required.

- For SRS, an invasive fixation using a stereotactic head frame can be used alternatively to image guidance.
- For SRS and FSRT, non-invasive fixation of the patient’s head is combined with image-guidance.
- For SBRT, image-guidance of the target itself (or a surrogate structure highly correlated with the target) is required. The optimal image-guidance strategy is dependent on the tumor site and location and needs to consider the following principles:
  - in cases of target motion relative to the bony anatomy, image-guidance requires volumetric imaging with or without implanted fiducial markers or electromagnetic transponders or requires stereoscopic X-ray imaging of the target itself or of implanted fiducial markers as target surrogate.
  - in cases at risk of serial organs-at-risk motion into areas of critical radiation doses, volumetric image-guidance is recommended.

#### (3) Motion management:

Systematic assessment and consistent consideration of periodic and non-periodic target motion during

- imaging for treatment planning;
- target volume definition;
- beam-delivery technique planning;
- dose simulation;
- target volume localization & repositioning; and
- dose application

using a time-resolved motion management strategy is required. Compensation of breathing-induced uncertainties can be performed using breath-hold technique, free-breathing with gated beam delivery, free-breathing with continuous beam delivery using the internal target volume (ITV) or mid-ventilation concept and free-breathing with dynamic tumor tracking.

#### (4) Collimation of the irradiation and beam directions:

For the respective treatment modalities, collimation and beam direction requires the following characteristics:

- SRS with multileaf collimator (MLC) with leaf width ≤5 mm or cylindrical collimators of equivalent size, both at normal treatment distance, and used with systems allowing non-coplanar beam directions.
- FSRT with MLC with leaf width ≤6.5 mm or cylindrical collimators of equivalent size, both at normal treatment distance.
- SBRT with MLC with leaf width <10 mm or cylindrical collimators of equivalent size, both at normal treatment distance.

However, for FSRT or SBRT close to radiation-sensitive critical structures the same collimation and beam direction requirements as for SRS are recommended.

#### (5) Dose calculation:

For stereotactic radiotherapy in areas with large density inhomogeneities the use of a dose calculation algorithm that takes into account lateral electron transport to correct for density inhomogeneities is required. The maximum grid size for dose calculation should be 1–2 mm according to the target lesion dimensions and the image resolution for target definition.

#### (6) Treatment unit accuracy:

A geometric accuracy with three-dimensional spatial dose placement in system-specific end-to-end tests requires inaccuracies of at maximum:

- 1 mm for SRS.
- 1.25 mm for FSRT and SBRT in non-moving phantoms.
- 1.5 mm for SBRT in moving phantoms.

However, for FSRT and SBRT close to radiation-sensitive critical structures the same geometric accuracy requirement as for SRS is recommended.

A dosimetric accuracy with point-based plan-to-measurement differences of maximum 3% within a target volume of more than or equal to 2 cc, measured in system-specific end-to-end or delivery-quality-assurance tests with homogeneous phantoms, is required. For target volumes smaller than 2 cc, the uncertainties of the measurement may be larger than the desired dosimetric accuracy.

#### (7) Dedicated quality assurance measures are required:

- Small field dosimetry for commissioning.
- System-specific end-to-end testing for both static and moving target volumes.
- Regular check of the geometric and dosimetric accuracy according to system-specific guidelines.
- Day-to-day quality control of the consistency of the stereotactic frame and/or the image-guidance system isocenter with the treatment beam isocenter.

### Process quality requirements

1. Written standard operating procedures for all stereotactic radiotherapy relevant process steps are required.
2. Interdisciplinary discussions on the indication for stereotactic radiotherapy are required.
3. A trained multiprofessional stereotactic radiotherapy project team (radio-oncology, medical physics, radiation therapists) for the implementation and application of SRS/FSRT/SBRT is required. Education and training should include:
  Participation in stereotactic radiotherapy specific training events (e.g., of the DEGRO, DGMP, ESTRO, ASTRO).
  Participation in stereotactic radiotherapy training by the corresponding industry partners of the clinical institution.
  Participation in hands-on practical training at centers experienced in stereotactic radiotherapy.
4. Sufficient experience in stereotactic radiotherapy with ≥20 patients treated each year with SRS, FSRT and SBRT (experiences gained in SRS can be transferred to FSRT) is required.
5. Prescribing, recording and reporting each SRS/FSRT/SBRT treatment procedure according to international guidelines and standards is required (for further details see the International Commission on Radiation Units and Measurements report 91 on prescribing, recording, and reporting of stereotactic treatments with small photon beams and the statement of the DEGRO/DGMP working group stereotactic radiotherapy and radiosurgery [10])
